# Systemic Multi-Omics Analysis Reveals Amplified P4HA1 Gene Associated With Prognostic and Hypoxic Regulation in Breast Cancer

**DOI:** 10.3389/fgene.2021.632626

**Published:** 2021-02-22

**Authors:** Manikandan Murugesan, Kumpati Premkumar

**Affiliations:** Department of Biomedical Science, School of Biotechnology and Genetic Engineering, Bharathidasan University, Tiruchirappalli, India

**Keywords:** breast cancer, hypoxia, prognosis, omics, computational biology

## Abstract

Breast cancer (BC) is a common malignant tumor in females around the world. While multimodality therapies exist, the mortality rate remains high. The hypoxic condition was one of the potent determinants in BC progression. The molecular mechanisms underpinning hypoxia and their association with BC can contribute to a better understanding of tailored therapies. In this study, two hypoxic induced BC transcriptomic cohorts (GSE27813 and GSE47533) were assessed from the GEO database. The P4HA1 gene was identified as a putative candidate and significantly regulated in hypoxic BC cells compared to normal BC cells at different time intervals (6 h, 9 h, 16 h, 32 h, and 48 h). In patients with Luminal (*p* < 1E-12), triple-negative subclasses (*p* = 1.35059E-10), Stage 1 (*p* = 8.8817E-16), lymph node N1 (*p* = 1.62436E-12), and in the 40–80 age group (*p* = 1.62447E-12), the expression of P4HA1 was closely associated with the clinical subtypes of BC. Furthermore, at the 10q22.1 chromosomal band, the P4HA1 gene displayed a high copy number elevation and was associated with a poor clinical regimen with overall survival, relapse-free survival, and distant metastases-free survival in BC patients. In addition, using BioGRID, the protein–protein interaction (PPI) network was built and the cellular metabolic processes, and hedgehog pathways are functionally enriched with GO and KEGG terms. This tentative result provides insight into the molecular function of the P4HA1 gene, which is likely to promote hypoxic-mediated carcinogenesis, which may favor early detection of BC and therapeutic stratification.

## Introduction

The second leading cause of tumor-related death worldwide is breast cancer (BC) (WCRF, 2018). Poly-etiology and the constituent nature of BC threaten early diagnosis and treatment strategies (Feng et al., 2018). BC is divided into five prevailing subtypes based on molecular profiling techniques: luminal A/B, basal-like, HER2(+), and normal breast-like. Molecular heterogeneity in BC inter-/intra-tumor also increases tumor growth and becomes more complex in therapy (Koren and Bentires-Alj, 2015; Haynes et al., 2017)A common trait of cancer cells is that they quickly proliferate, consuming significant amounts of oxygen that hampers the low-level oxygen state called hypoxia. The hypoxia-inducible factor 1 (HIF-1) regulator pathway gets activated once the cancer cell enters hypoxic conditions (1–5% O_2_), contributing to the promotion of angiogenesis and metastatic tumor characteristics in BC (Murugesan and Premkumar, 2018; Al Tameemi et al., 2019; Dillekas et al., 2019). In invasive-BC tumors, about 50–60% with hypoxic regions and suggests a critical determinant of metastasis (Greer et al., 2012). Almost 90% of BC deaths are reported due to delayed late diagnosis (Dillekas et al., 2019). Clinical studies show that hypoxia is one of the primary drivers of epithelial-mesenchymal transformation (EMT) and metastatic cascade transition (Dillekas et al., 2019). In addition, HIF-1 was implicated in hematogenous breast metastases to lung cancer and was associated with low patient survival and resistance to chemotherapy in breast (Campbell et al., 2019), gastric (Cheng et al., 2012), and colorectal (Baba et al., 2010) cancer.

The accumulating knowledge in microarray databases (Oncomine, GEO, Array Express, and so on) using genome-wide technologies has played an essential role in exploring the cancer-related molecular pathogenesis portfolio (Siegel et al., 2018; Ha et al., 2019; Shou et al., 2020; Yang et al., 2020). In future contexts, the ability to dissect and incorporate cancer omics data opens the door to a new approach to the biomarker strategy for diagnosis and treatment. In the same way, TCGA provides a multi-cancer cohort of RNA-Seq transcriptomics, which has led to a significant increase in understanding the biology of malignancy. Its accessibility has led to a splendid opportunity to extend molecular tumors’ fundamental mechanisms (Manzoni et al., 2018).

Prolyl collagen 4-hydroxylase (P4H) is a tetrameric α2β2 α-ketoglutarate (α-KG) –dioxygenase that is responsible for collagen folding and stabilization. Collagens, which are the most abundant proteins in humans, provide extracellular matrix (ECM) assembly scaffolding (rigidity and cell adhesion) (Koski et al., 2017) and are also associated with stabilizing tumor proliferation (Provenzano et al., 2006). Three P4HA isoforms in mammalian cells (P4HA1-3) were identified. Of the three isoforms, P4HA1 is the foremost isoform that contributes to the foremost peptide bond and protein scaffolding activity. P4HA2 is also involved in the collagen synthesis and folding of collagen chains. The P4HA1 is majorly expressed in the testis and placenta, P4HA2 in adipose tissue, and P4HA3 in the heart and placenta. Reports suggest P4HA1 and P4HA2 to be associated with cancer proliferation and hypoxic regulation (Weinschenk et al., 2002; Cioffi et al., 2003; Kukkola et al., 2003; Willam et al., 2006; Gorres and Raines, 2010). In addition, P4HA1 enhances EMT and stemness of malignant cells through the HIF-1 pathway (Kappler et al., 2017; D’Aniello et al., 2019). P4HA1 has recently been found to overexpress in gliomas and HNSCC; its expression associated with tumor microvessel density (Li et al., 2019). Recent studies have shown that increased production of collagen is linked to BC progression, adhesion, and invasion (Xiong et al., 2018; Wishart et al., 2020).

However, the potential effects of P4HA1 and their precise contribution to BC are not entirely explored. This research extensively examined the expression of P4HA1 in breast cancer cells and its therapeutic relevance in tumor-affected samples using integrative functional multi-omic approaches. In addition, the regulatory genes of P4HA1 and their molecular, pathological, and signaling predictive role in BC consented. In a diagnostic and treatment regimen to control BC malignancy, P4HA1 could be an effective target.

## Materials and Methods

### Microarray Data

**FIGURE 1:**
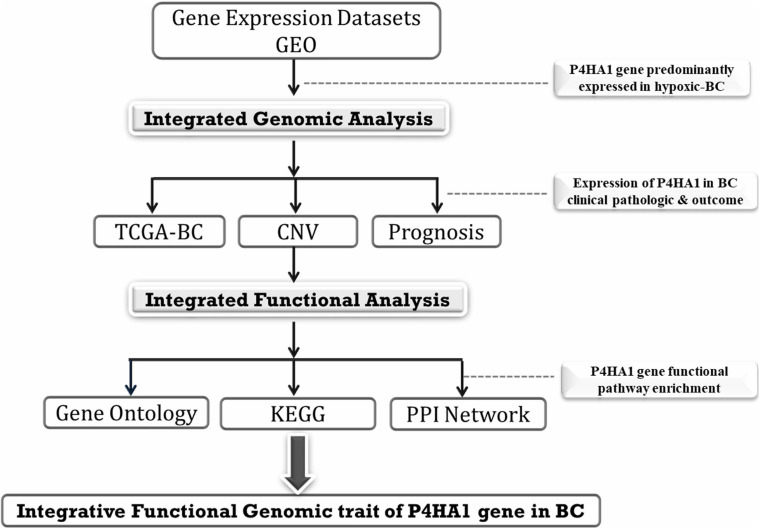
A flow chart of integrated functional analysis.

The GSE27813 and GSE47533 transcriptomic profiles of breast cancer cells subjected to hypoxia conditions (1% O2) were downloaded from the Gene Expression Omnibus (GEO) database^1^ of the National Center for Biotechnology Information (NCBI) and explored in the current study. The studies were carried out on two different platforms GPL10558-Illumina Human HT-12 V4.0 bead chip expression and GPL6884-Illumina Human WG-6 v3.0 bead chip expression. The normalized data were downloaded, and probes were annotated with authentic gene symbols from each platform using the required Illumina annotation files. Integrative analysis of these BC mRNA transcriptomes with/without hypoxic exposure profiles was used to identify the potential genes at various time intervals. The full integrated analysis chart had shown in Figure 1.

### The Cancer Genome Atlas (TCGA) Data Validation

TCGA is a web-based platform that visualizes, integrates, and analyses malignancy genomics and associated clinical results. UALCAN^2^ can be an intuitive, user-friendly, open-source web portal for an in-depth study of TCGA data (Chandrashekar et al., 2017). UALCAN uses RNA-Seq level 3 of TCGA and clinical data on 31 cancer types. The expression of the candidate gene in normal tissues was subsequently weighed against the corresponding BC tissues. Moreover, overall survival (OS)/recurrence-free survival (RFS) was assessed using Kaplan–Meier survival curves, and the hazard ratio (HR) was determined with 95% confidence intervals, and log-rank *p*-value was ascertained. Furthermore, assessment of mRNA expression of P4HA1 among different subtypes of breast tumors was achieved to explore the pathological characteristics of genes in tumor initiation or progression.

### Oncomine Database Analysis

The expression level of P4HA1 was derived from the oncomine database^3^ in various BC transcriptomic profiles. The oncomine interface (Compendia biosciences, Ann Arbor, MI, United States) is an online archive of previously published, open-access microarray data widely distributed and freely accessible to cancer repositories (Rhodes et al., 2004). The differential expression of mRNA in cancer tissue relative to normal was achieved using the parameters of the *p*-value threshold of 0.01 and fold-change (FC) > 2.

### Copy Number Alteration Analysis

Using Progenetix (Progenetix, Stanford, CA, United States)^4^, DNA copy number variations (CNVs), such as deletions and amplification in BC transcriptomic cohorts, were examined (Baudis and Cleary, 2001). It is an online repossession of cancer molecular-cytogenetic data that captures the robust, best-grained understanding of the absolute copy number aberration. The chromosomal variation features of the P4HA1 gene were analyzed in the TCGA-BC data to produce frequency gain/loss.

### Clinical Regimen Prognosis

Kaplan–Meier Plotter^5^ is a data source that integrates gene expression and clinical data on about 21 cancer types, including breast cancer (*n* = 6234) (Gyorffy and Schafer, 2009). KM Plotter was used to study the prognosis value for P4HA1 in BC. We centered our assessment on the overall patient survival (OS), distance metastasis-free survival (DMFS), and relapse-free survival (RFS). The log-rank *p*-value and hazard ratio with 95% confidence intervals additionally ascertained. The Cox proportional hazard regression model with microarray cohort GSE22133 was examined to verify the patient’s overall survival between the expression of the P4HA1 gene and the BC’s clinical characteristics. The median P4HA1 value is the threshold used to evaluate the prognostic score of each parameter.

### Protein–Protein Network

Protein–protein interaction networks provide information on the molecular framework of cellular processes and integral mobile activity. In the present research, a PPI network of P4HA1 regulatory genes built using an online database, the Biological General Repository for Interaction Datasets (BioGRID) v3.5.175^6^, a database of already established networks; incorporates 1,728,498 protein and genetic interactions (Oughtred et al., 2019). In the BioGRID database, we have imported the lists of co-expressed P4HA1 genes. To create and visualize the PPI network for the P4HA1 protein, Cytoscape v3.5.1 was employed. The PPI network’s primary nodes were then grouped according to the enrichment of the KEGG Pathway. Hub nodes with a higher degree would be in phase to delineate their significant role in the BC progression.

### Pathway Enrichment Analysis

We conducted pathway enrichment (GO and KEGG) using g:Profiler^7^ to explore the function of P4HA1 gene sets with biochemical, cellular, and molecular aspects (Raudvere et al., 2019). g:Profiler searches for a collection of the pathway, network, regulatory motif, and phenotype gene sets using a detailed set of accurate and concise annotation methods. The method also consolidates the exact Fisher test with an input gene list and *p*-value enrichment for each pathway. Using a threshold of 0.05, the g:Profiler computes the *p*-values from GO and KEGG route enrichment analysis.

### Statistical Analysis

The transcriptomic cohort analysis was performed using the R programming environment (version 3.2.5) with the criteria of *p*-value < 0.05. Survival analysis was conducted jointly with Kaplan–Meier plots and COX Proportional hazard model. The Kaplan–Meier curves were used to assess the overall survival, relapse-free survival, and distance metastasis-free survival associated with the P4HA1 gene expression. The univariate and multivariate Cox proportional model was carried to analyze the association of P4HA1 with the clinicopathologic variants of breast cancer and estimate the hazard ratio and 95% CIs. Logistic regression analysis was carried out in GSE22133 data to explore the association of P4HA1 gene expression with the clinicopathologic variants of breast cancer: ER, PR, and Grade. It estimates the breast cancer risk by examining the odds ratios (ORs) and 95% confident intervals (CIs), and *p*-value. The two-tailed *p*-values below 0.05 were considered statistically significant.

## Results

### P4HA1 Expression in BC Under Hypoxic Condition

**FIGURE 2:**
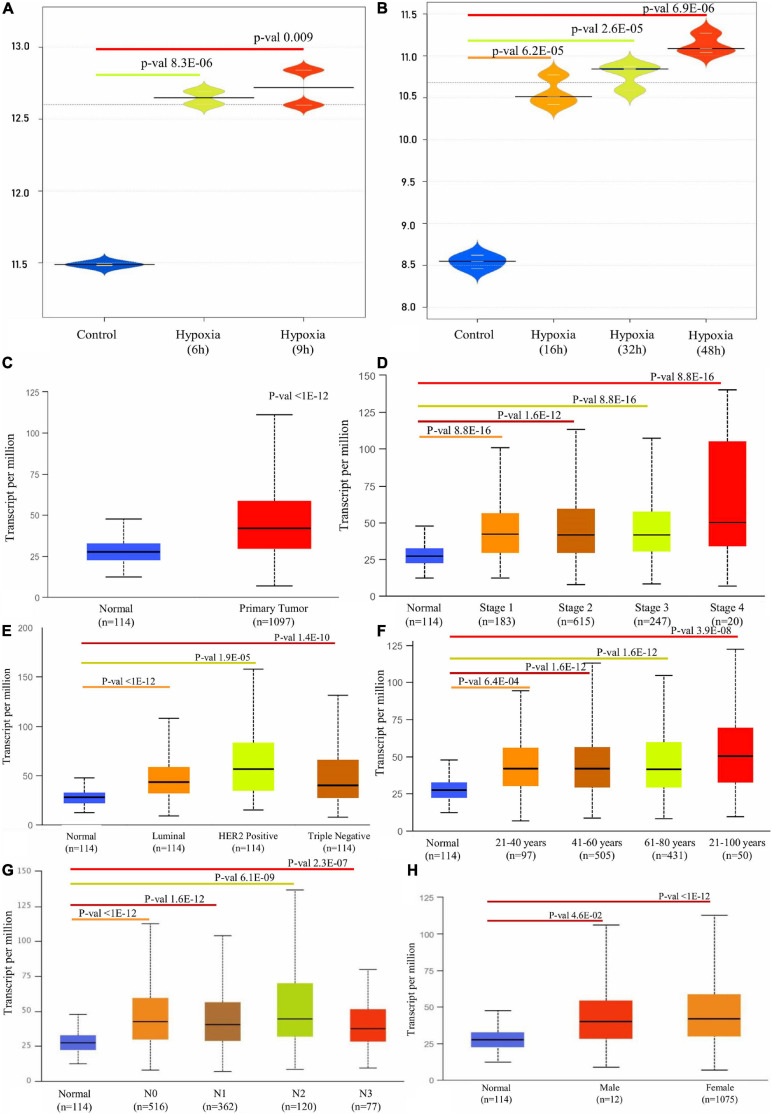
Box plot representation of P4HA1 gene expression compared with a normal and different time period of hypoxic exposure in BC cells: cut-off *p*-value < 0.05. **(A)** GSE27813 and **(B)** GSE47533. **(C–H)** Box plot showing relative expression of P4HA1 in clinicopathologic of Breast Cancer, **(C)** Normal and Primary Tumor samples, **(D)** Normal and patients in Stages 1, 2, 3, and 4, **(E)** Normal and Subclass, **(F)** Normal and Age group, **(G)** Normal and Nodal subclass, and **(H)** Normal and Gender.

A detailed description of the transcriptomic data used in this study was given in Table 1. An integrative analysis of these cohorts identified a high-expression P4HA1 gene with a p-threshold criterion of <0.05 and FC > 2 in the two datasets. Moreover, P4HA1 was remarkably increased during the different time (6 h, 9 h, 16 h, 32 h, and 48 h) of the hypoxic state. The Violin Plot revealed the difference between with and without hypoxic exposure in breast cancer cells in the mRNA expression of P4HA1 (Figures 2A,B).

**TABLE 1:** Characteristics of transcriptomic data from Gene Expression Omnibus.

GEO ID	Platform Acc.	Platform	Cell line	Time period of hypoxia (1% O_2_)	Year
*GSE27813*	GPL10558	Illumina Human HT-12 v4.0 Expression BeadChip	MCF-7	6 h, 9 h	2011
*GSE47533*	GPL6884	Illumina HumanWG-6 v3.0 Expression BeadChip	MCF-7	16 h, 32 h, 48 h	2014

### Transcriptional Expression of P4HA1 in the Clinical Regimen of BC

A differential transcriptional level of P4HA1 between BC and paired normal breast tissue was evaluated by the UALCAN database to determine the mRNA expression of P4HA1 in BC patients. As illustrated in Figure 2, the transcriptional level of P4HA1 was substantially up-regulated in BC tissues (Figure 2C, *p* ≤ 1E-12) compared to normal tissues. Subsequently, P4HA1 differential transcriptional levels were compared for the molecular and histological subtypes, tumor grades, and other BC patient factors. Box plots were made to visualize the association between the expression levels of the clinicopathologic condition of BC. As shown in Figure 2, the level of P4HA1 was significantly associated with the intrinsic subclasses of the BC. Patients with Luminal (*p* ≤ 1E-12) and triple-negative subclasses (*p* = 1.35059E-10) tend to express a higher P4HA1, than HER2-positive (*p* = 1.9099E-05). The highest mRNA expressions of P4HA1 were found sequentially in the various stages of the BC, Stage 1 (*p* = 8.8817E-16) <Stage 3 (*p* = 1.670441E-12) <Stage 2 (*p* = 1.62447E-12) <Stage 4 (*p* = 1.31617E-03) (Figure 2D), and the highest mRNA expressions of P4HA1 were similarly found in-between the age group of 40–80 (*p* = 1.62447E-12) and marginally lower in age <80 (*p* = 3.9105E-08) than the >40 (*p* = 6.3915E-04) age group (Figure 2F). Interestingly, P4HA1 expression was analyzed with the metastatic lymph node classification and elevated level of expression in N1 (*p* = 1.62436E-12) than N0 (*p* ≤ 1E-12) <N2 (*p* = 6.06390E-09) <N3 (*p* = 2.31799E-07) (Figure 2G). Together, the results showed a positive association between P4HA1 transcriptional levels and typical subclasses in BC patients.

**FIGURE 3:**
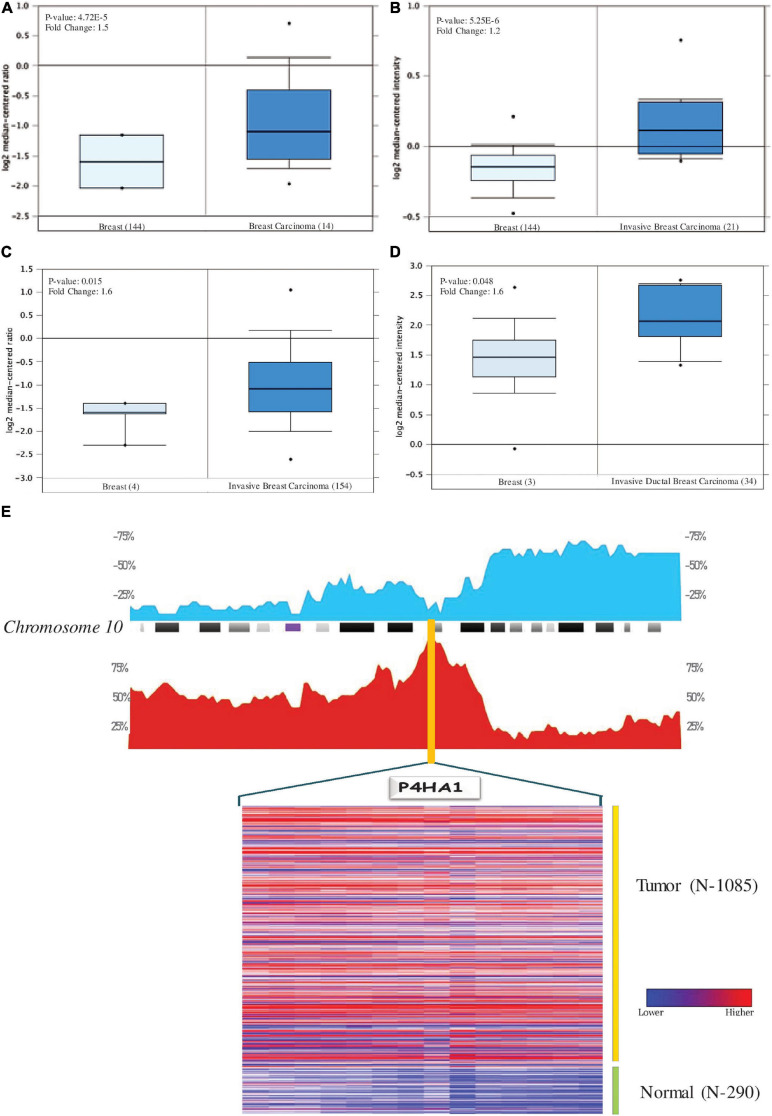
Levels of P4HA1 mRNA expression in BC compared to normal cells. Figures generated based on Oncomine analysis with criteria fold-change and *p*-values. **(A)** Zhao Breast, **(B)** Curtis Breast, **(C)** Gluck Breast, and **(D)** Curtis Breast Dataset. **(E)** The distribution of Copy number variation of schematic physical map of Chromosome 10 (human genome 19 assembly (GRCh37) for TCGA Breast carcinoma generated from Progenetix tool. Heat map representation of P4HA1 between the normal and breast cancer patients – TCGA data. The color ratio red to green represents the change from high to low.

Oncomine analysis of malignant breast tissue relative to normal tissue analysis showed altered expression of P4HA1 in different transcriptomic profiles (Figure 3). In the Curtis data set, the P4HA1 mRNA rate was significantly higher in the breast tumor (FC = −1.570, *p* = 4.72E-5). Furthermore, in invasive breast carcinoma, there was a substantial rise in mRNA levels of P4HA1 (FC = 1.219, *p* = 5.25E-6). Moreover, P4HA1 was up-regulated in the Gluck (FC = 1.641, *p* = 0.015) and Zhao (FC = 1.598, *p* = 0.048) datasets.

### P4HA1 Genomic Alteration in BC

With genome-wide copy number profiles in the Progenetix database, we investigated the prevalent genomic amplification of the P4HA1 chromosomal region in BC. We focused on the use of the TCGA-BC cohort and obtained a recurring functional copy number gain for chromosome 10q22.1 (location P4HA1) (Figure 3E). Since this is the typical copy number peaks in cancers, it can aid BC’s development and metastatic niche.

### Prognostic Significance of P4HA1 in BC

**FIGURE 4:**
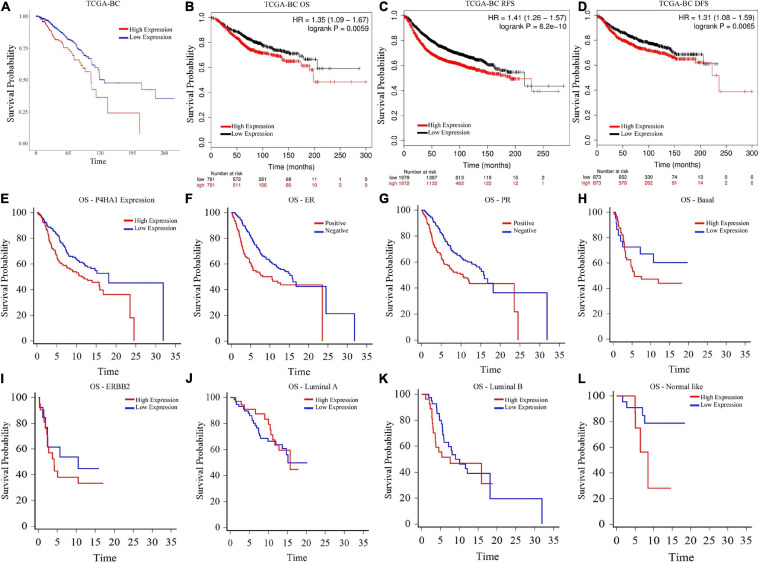
Prognostic Index of Breast cancer as determined by Kaplan–Meier estimates **(A)** Overall Survival (UALCAN), **(B)** Overall Survival (Kaplan–Meier Plotter), **(C)** Relapse-free survival (Kaplan–Meier Plotter), **(D)** Distant free metastasis survival (Kaplan–Meier Plotter), **(E)** Overall Survival-P4HA1 Expression (GSE22133), **(F)** Overall Survival-Estrogen Receptor (GSE22133), **(G)** Overall Survival-Progesterone Receptor (GSE22133), **(H)** Overall Survival-Basal (GSE22133), **(I)** Overall Survival-ERBB2 (GSE22133), **(J)** Overall Survival-Luminal A (GSE22133), **(K)** Overall Survival-Luminal B (GSE22133), and **(L)** Overall Survival-Normal-like (GSE22133).

**FIGURE 5:**
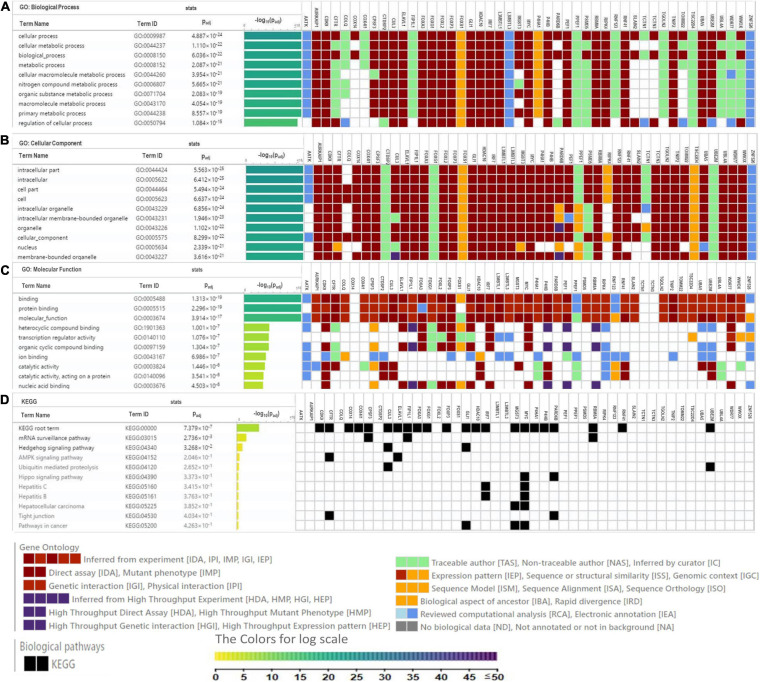
Heat map depicts the associations of P4HA1 signature with GO term and KEGG generated by g:Profiler. **(A)** GO-Biological Process, **(B)** GO-Cellular Component, **(C)** GO-Molecular Function, and **(D)** KEGG pathway.

To evaluate the clinical significance of P4HA1 with BC, we analyzed the patient’s survival index through the Kaplan–Meier plotter and UALCAN (Figure 4). The regulation of P4HA1 significantly contributes to the worst prognostic in BC patients. OS was significantly shorter in patients with elevated P4HA1 (HR = 1.35; 95% CI: 1.09–1.67; *p* < 0.0059) (Figures 4A, 5B) compared to low P4HA1 expression. Moreover, the higher expression of P4HA1 indicated poor RFS (HR = 1.41; 95% CI: 1.26–1.57; *p* < 6.2E-10) (Figure 4C) and DMFS (HR = 1.31; 95% CI: 1.08–1.59; *p* < 0.0065) (Figure 4D). These findings show that P4HA1 is critically associated with a poorer clinical regimen in BC patients.

A univariate and multivariate regression analysis of Cox hazard regression using GSE22133 data was explored to verify the prognostic index of P4HA1. The association risk was estimated with the clinicopathologic covariates, including estrogen receptor (ER), progesterone receptor (PR), histological subtypes, and grades. Table 2 shows how the P4HA1 gene is associated with clinical factors. Univariate Cox regression analysis indicated a significant association with hormonal receptor ER (*p* = 0.0042, HR = 0.62, 95% CI = 0.46-0.86), PR (*p* = 0.0043, HR = 0.63, 95% CI = 0.46-0.86), and Grade (*p* = 0.051, HR = 1.21, 95% CI = 0.99-1.48) in GSE22133 data. In addition, multivariate Cox analysis found no strong association between histological subtypes and hormone receptors. Each clinical factor’s survival plot was depicted in Figures 4E–L. These results indicate that the P4HA1 expression strongly attributes to the hormonal receptor, ER, and PR.

**TABLE 2:** Univariate and multivariate analysis of clinicopathological factors associated with the prognostic significance of P4HA1 expression in breast cancer.

Clinical factors	GSE22133					
	Univariate			Multivariate		
	*p*-value	HR	CI (95%)	*p*-value	HR	CI (95%)
**P4HA1 expression**	0.0097	1.51	1.10–2.07	0.1693	1.34	0.88–2.04
**ER status** Positive vs. Negative	0.0042	0.62	0.45–0.86	0.8055	0.92	0.51–1.66
**PR status** Positive vs. Negative	0.0043	0.63	0.46–0.86	0.5944	0.86	0.49–1.48
**Grade** 1 and 2 vs. 3	0.0531	1.21	0.99–1.48	0.3255	1.12	0.88–1.43
**Histological subtypes** Basal ERBB2 Luminal A Luminal B Normal-Like	0.2847 0.4776 0.7437 0.4840 0.0880	1.53 1.39 0.88 1.26 3.41	0.70–3.34 0.56–3.45 0.43–1.79 0.65–2.44 0.83–14.00		NA	

*ER, Estrogen Receptor; PR, Progesterone Receptor; CI, Confidence Interval.*

Table 3 shows the logistic regression analysis of the association between the P4HA1 expression and clinicopathologic variants of breast cancer (ER, PR, and Grade). The expression of P4HA1 was significantly associated with the ER status group of breast cancer (OR = 0.38; 95% CI: 0.79–0.80, *P* = 0.011) but less significantly associated with the PR status group cancer (OR = 1.47; 95% CI: 0.71–3.03, *P* = 0.29). We assessed the association of P4HA1 expression with breast cancer grade through combing grade 1 and grade 2 vs. grade 3 and results revealed no significance associated with grades (OR = 1.40; 95% CI: 0.76–2.57, *P* = 0.27). In addition, this analysis also revealed a strong association of P4HA1 gene expression with the ER of breast cancer.

**TABLE 3:** Logistic regression analysis of associations between P4HA1 expression and the clinicopathologic variants of breast cancer.

Variable		Size	*P*-value	Odds ratio	95% CI
ER	Pos (173) vs. Neg (173)	346	0.0118	0.3811	0.1799 to 0.8076
PR	Pos (172) vs. Neg (171)	343	0.2964	1.4718	0.7126 to 3.0399
Grade	1 and 2 (116) vs. 3 (116)	232	0.2730	1.4035	0.7656 to 2.5731

*ER, Estrogen Receptor; PR, Progesterone Receptor; CI, Confidence Interval.*

### Biological Interaction of P4HA1

Gene Ontology (GO) analysis was carried out against using P4HA1 and its associated genes generated from the BioGRID source. We applied a hypergeometric test for each enriched GO term, with a threshold lower than 0.05 in the g:Profiler tool: (Figure 5). Under the GO hierarchy, the ontology of highly enriched biological processes was “Cellular Process” (GO:0009987), “Cellular Metabolic Process” (GO:0044237). In cellular ontology, the enriched terms were “intracellular part” (GO:0044424) and, similarly, with the ontological molecular function “Binding” (GO:0005488), were highly enriched (Table 4). Apart from the significant enrichment of the Kyoto Encyclopedia of Genes and Genomes (KEGG) pathway terms were the “mRNA surveillance pathway,” “Hedgehog signaling pathway,” and “AMPK signaling pathway.” The full enrichment analysis output is listed in Supplementary Table 1 (GO) and Supplementary Table 2 (KEGG). Most critically, many of these genes are associated with cellular metabolic shift and oncogenic signaling pathways, a process intimately linked with invasion and proliferation.

**TABLE 4:** Functional enrichment pathway analysis: Top enriched terms of gene ontology-biological process, cellular component, molecular function, and KEGG pathways.

Source	Term Id	Term name	*p*-value
***Gene ontology-biological process***			
*GO:BP*	GO:0009987	cellular process	4.89E-24
*GO:BP*	GO:0044237	cellular metabolic process	1.11E-22
*GO:BP*	GO:0008150	biological_process	6.04E-22
*GO:BP*	GO:0008152	metabolic process	2.09E-21
*GO:BP*	GO:0044260	cellular macromolecule metabolic process	3.95E-21
***Gene ontology-cellular component***			
*GO:CC*	GO:0044424	intracellular part	5.56E-25
*GO:CC*	GO:0005622	intracellular	6.41E-25
*GO:CC*	GO:0044464	cell part	5.49E-24
*GO:CC*	GO:0005623	cell	6.64E-24
*GO:CC*	GO:0043229	intracellular organelle	6.86E-24
***Gene ontology-molecular function***			
*GO:MF*	GO:0005488	binding	1.31E-19
*GO:MF*	GO:0005515	protein binding	2.30E-19
*GO:MF*	GO:0003674	molecular_function	3.91E-17
*GO:MF*	GO:1901363	heterocyclic compound binding	1.00E-07
*GO:MF*	GO:0140110	transcription regulator activity	1.08E-07
***KEGG***			
*KEGG*	KEGG:03015	mRNA surveillance pathway	0.002436223
*KEGG*	KEGG:04340	Hedgehog signaling pathway	0.002867797
*KEGG*	KEGG:04152	AMPK signaling pathway	0.002946176
*KEGG*	KEGG:04120	Ubiquitin mediated proteolysis	0.003652434

### Protein Interaction Network of P4HA1

**FIGURE 6:**
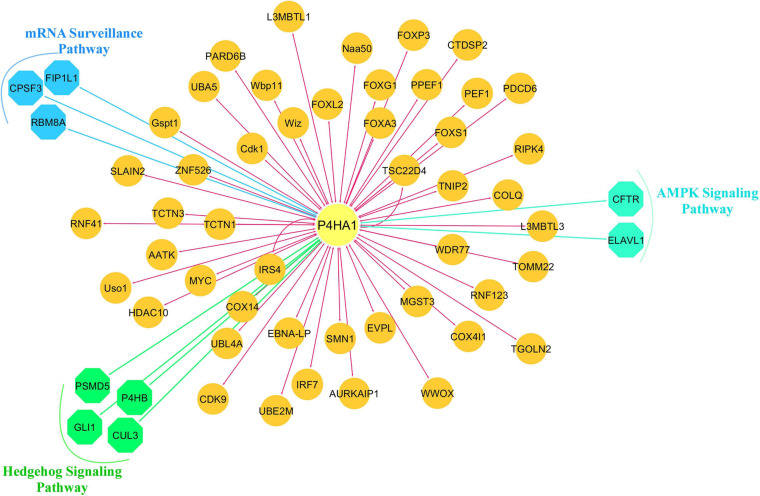
Protein-protein interaction network of P4HA1. Highly interacted protein network generated from the BioGRID source. Different colors of the network edge indicate functional enrichment with the KEGG database.

We constructed a P4HA1 mRNA interaction network generated from the BioGRID database. The final PPI network generated by Cytoscape consisted of 59 nodes and 382 interactions (Supplementary Table 3). Each signaling pathway’s proteins were colored based on the KEGG enrichment (Figure 6).

## Discussion

Breast cancer heterogeneity is still one of the most frequent causes of cancer mortality (Lin L. F. et al., 2019). Despite multimodal care for patients, the hypoxic condition is a critical factor that affects the treatment strategy and the clinical regimen (Tong et al., 2018). The knowledge in genotypical and their profound mechanisms will also advance the effective therapeutic stratification of BC. Microarray and next-generation (NGS) sequencing methods have recently been used for early detection and personalized treatment (Wang et al., 2009; Marzancola et al., 2016). Such diverse data offers an outstanding opportunity to discuss more concerns relevant to tumor heterogeneity. A compendium of an integrative functional approach was systematically proposed to explore the P4HA1 gene fundamentally associated with hypoxia-induced BC. To delineate the processes involved in carcinogenesis, the reliability of this analysis was validated in terms of expression, clinical subtypes, copy number variation, and altered pathways in the clinical TCGA-BC cohort. Therefore this analysis merged transcriptional activities with molecular signaling pathways to underpin the proliferation of hypoxic-mediated BC.

Our findings revealed that P4HA1 gene expression is reliably expressed in breast cancer vs. normal cells. It was consistently noted in BC subclasses, that in patients with Luminal, triple-negative, and lymph node (N1), P4HA1 was overexpressed but comparatively lower in the positive HER2 group and P4HA1 was prominent in Stage I compared to the other BC stages. Overexpression of P4HA1 has previously been seen in TNBC-BC (Xiong et al., 2018), head and neck squamous cell carcinomas (HNSCC) (Li et al., 2019), prostate (Wolf et al., 2004), melanoma (Atkinson et al., 2019), and gastric cancer (Cheng et al., 2012). Importantly, our study showed that overexpression of P4HA1 could be associated with tumor progression, invasion and thus act as a diagnostic biomarker of BC.

A distinctive molecular mechanism explains the strong association between CNV and differential expression of P4HA1. We observed that the P4HA1-10q22.1 copy number showed a high-level positive amplification in the patient data for TCGA-BC, suggesting its effect on the high mRNA transcription level. Moreover, the association in the elevated amplicon 10q22 was reported to have a remarkable role in tumorigenesis and weak prognostic significance in patients with prostate cancer (Wolf et al., 2004), gastric cancer (Cheng et al., 2012), glioma (Hu et al., 2017), melanoma (Atkinson et al., 2019), oral squamous cell carcinoma (Kappler et al., 2017), and HNSCC (Li et al., 2019). In line with previous studies, higher P4HA1 expression was also directly related to BC patients’ poor survival and could be accomplished as a prognostic predictor.

Functional enrichment analysis of gene ontology revealed that genes were mostly involved in different cellular metabolic processes. Most frequently, by increased glycolytic flux and suppressed oxidative phosphorylation (Warburg effect), tumor cells adapt their resources to cope with high energy demands. Thus, the hypoxic state acquires energy via the hypoxic receptive elements (HRE) through the metabolic shift and tumor microenvironment (Dillekas et al., 2019). Under physiological oxygen concentrations, Prolyl hydroxylase (PHD1-3) enzymes strengthen the stability of HIF1 and HREs. Previous studies have shown that PHD enzymes involved in HRE’s regulatory network in gastric cancer and PHD inhibition contribute to reduced tumor development under hypoxic conditions (Cheng et al., 2012). Interestingly, the presence of PHDs is closely related to tumor angiogenesis and metastasis during hypoxic cell proliferation.

We observed that the F gene and the RBM8A gene were closely associated with an mRNA surveillance pathway in the KEGG pathway enrichment. The Cleavage polyadenylation specificity factor (CPSF) is a multi-subunit that actively participates through the cleavage and polyadenylation of mRNA activation in the eukaryotic pre-messenger (m)RNA 3′-end process (Casanal et al., 2017). Importantly, these CPSF factors lead to the growth of human cancer, such as breast (Erson-Bensan and Can, 2016), ovarian cancer (Zhang et al., 2017), and even the inhibition of CPSF3 actuates apoptosis in prostate cancer cells (Van Etten et al., 2017). Interestingly, CPCF3 and CPCF4 were a major component of the OS and RFS based CPSF complex in non-small lung cancer (Ning et al., 2019).

RNA binding motif protein 8A (RBM8A), also known as Y14, is an essential factor in exon junction complex (EJC), translation, chromatin remodeling, damage checkpoints, regulation of apoptosis (Gerstberger et al., 2014), and deregulation contribute to cancer pathologies and cardiovascular diseases (Wurth and Gebauer, 2015). RBM8A up-regulation is critically involved in modulating apoptosis, and tumor proliferation and metastasis (Lu et al., 2017). Cell growth was blocked in RBM8A knockout cells, and the G2/M step of the cell cycle was arrested in lung adenocarcinoma cells (Ishigaki et al., 2013). In addition, for individuals with hepatocellular carcinoma, elevated RBM8A expression was associated with poor prognosis and progression-free survival. RBM8A tends to be active in the EMT transition, an important occurrence in the metastatic niche (Lin Y. et al., 2019).

Hedgehog signaling (Hh) plays a vital role in embryonic cellular differentiation, and its alteration has oncogenic functions in initiating and progression (Sari et al., 2018; Chang and Lai, 2019). One of the downstream regulators of the Hh route was the Cullin gene. Cullin 3 proteins are active in cell cycle regulation and redox homeostasis, protein trafficking, and stress responses (Chen and Chen, 2016). Interestingly, CUL3 up-regulation is associated with an acquired carcinogenic state and oxidative stress in BC (Loignon et al., 2009). Recent evidence indicates that Cullin-dependent ubiquitin ligases play a crucial role in breast carcinogenesis and squamous cell carcinoma of the esophagus (Hu et al., 2018).

Glioma-associated oncogene transcription factors (GLI) is a Zinc finger protein and downstream regulator of the Hh pathway (Pietrobono et al., 2019). In early embryonic development, GLI members play a major role in the central nervous system; however, it is also involved in carcinogenesis and metastatic cascade niche (Niewiadomski et al., 2019). Since amplified GLI was first observed in glioblastoma, it has now been commonly detected in the breast (Song et al., 2016), lung (Panneerselvam et al., 2019), pancreatic (Kowolik et al., 2019), colorectal (Park et al., 2019), leukemia (Jetten, 2019), and renal cell carcinoma (Kotulak-Chrzaszcz et al., 2019). It was also stated that high-expression GLI prevails tumor suppression mediated by p53 (Abe et al., 2008). Silencing GLI decreases cancer cell proliferation and invasive potency (Mishra et al., 2019). These results indicate a mechanism of Hh signaling to stimulate malignant stemming and facilitate the growth of tumors.

## Conclusion

This study used robust multiple transcriptomic cohorts with an integrated omic analysis and found that P4HA1 may be a potential oncogenic biomarker in BC. Moreover, this gene showed a copy number gain, reliably more explicit in high-grade metastatic breast tumors with poor clinical patient results. Besides, we speculate the implication of the hedgehog signaling pathway and metabolic reprogramming during high cell proliferation in hypoxic breast tumors. Our studies have provided useful insights into the P4HA1; it can be a novel biomarker for the diagnosis and progression of BC therapy.

## Data Availability Statement

This study was carried out on publicly available data on Gene Expression Omnibus (https://www.ncbi.nlm.nih.gov/geo/) with accession numbers: GSE27813, GSE47533, and GSE22133.

## Author Contributions

MM and KP conceived and designed the study. MM performed the integrated analysis, acquired the data, and drafted the manuscript. KP assisted with reviewing and editing the manuscript. Both authors approved the final manuscript for publication.

## Conflict of Interest

The authors declare that the research was conducted in the absence of any commercial or financial relationships that could be construed as a potential conflict of interest.

## Footnotes

1 https://www.ncbi.nlm.nih.gov/gds/

2 http://ualcan.path.uab.edu/index.html

3 https://www.oncomine.org/resource/login.html

4 http://www.progenetix.net

5 http://kmplot.com/analysis/

6 http://thebiogrid.org/

7 http://biit.cs.ut.ee/gprofiler/
